# IBRORS-MCL study: a Spanish retrospective and observational study of relapsed/refractory mantle-cell lymphoma treated with ibrutinib in routine clinical practice

**DOI:** 10.1007/s12185-022-03367-z

**Published:** 2022-05-13

**Authors:** Juan-Manuel Sancho, Ana Marín-Niebla, Silvia Fernández, Francisco-Javier Capote, Carolina Cañigral, Carlos Grande, Eva Donato, Izaskun Zeberio, Jose-Manuel Puerta, Alfredo Rivas, Elena Pérez-Ceballos, Ana Vale, Alejandro Martín García-Sancho, Antonio Salar, Eva González-Barca, Anabel Teruel, Carmen Pastoriza, Diego Conde-Royo, Joaquín Sánchez-García, Cristina Barrenetxea, Reyes Arranz, José-Ángel Hernández-Rivas, María-José Ramírez, Aroa Jiménez, Eva Rubio-Azpeitia

**Affiliations:** 1 Clinical Hematology Department, Catalan Institute of Oncology (ICO), ICO Hospital Germans Trias i Pujol, Carretera de Canyet, s/n, 08916 Badalona, Barcelona Spain; 2 Hospital Universitario Vall d’Hebron, Passeig de la Vall d’Hebron, 119-129, 08035 Barcelona, Spain; 3 Hospital de León, Altos de Nava, S/N, 24071 León, Spain; 4 Hospital Puerta del Mar, Av. Ana de Viya, 21, 11009 Cádiz, Spain; 5 Hospital de Castellón, Av. de Benicàssim, 128, 12004 Castellón de la Plana, Castellón Spain; 6 Hospital Doce de Octubre, Av. de Córdoba, s/n, 28041 Madrid, Spain; 7 Hospital Universitario Dr. Peset, Av. de Gaspar Aguilar, 90, 46017 Valencia, Spain; 8 Hospital Donostia, Begiristain Doktorea Pasealekua, s/n, 20014 Donostia, Gipuzkoa Spain; 9 Hospital Virgen de Las Nieves, Av. de las Fuerzas Armadas, 2, 18014 Granada, Spain; 10 Hospital Clìnic i Provincial, C. de Villarroel, 170, 08036 Barcelona, Spain; 11 Hospital Morales Meseguer, Av Marqués de los Vélez, s/n, 30008 Murcia, Spain; 12 Complejo Hospitalario Universiatrio A Coruña, As Xubias, 84, 15006 A Coruña, Spain; 13 Hospital Universitario de Salamanca, IBSAL, CIBERONC, Paseo de San Vicente 182, 37007 Salamanca, Spain; 14 Hospital del Mar, Passeig Marítim de la Barceloneta, 25, 29, 08003 Barcelona, Spain; 15 Hospital Duran i Reynals, Av. de la Granvia de l’Hospitalet, 199,, 08908 L’Hospitalet de Llobregat, Barcelona Spain; 16 Hospital Clínico Universitario de Valencia, Av. de Blasco Ibáñez, 17, 46010 Valencia, Spain; 17 Hospital de Orense, Ramón Puga Noguerol, 54, 32005 Orense, Spain; 18 Hospital Príncipe de Asturias, Carr. de Alcalá, s/n, 28805 Meco, Madrid Spain; 19 Hospital Reina Sofia, IMIBIC, UCO, Av. Menendez Pidal, s/n, 14004 Córdoba, Spain; 20 Hospital Basurto, Montevideo Etorb., 18, 48013 Bilbo, Bizkaia Spain; 21 Hospital la Princesa, Diego de León, 62, 28006 Madrid, Spain; 22 Hospital Universitario Infanta Leonor, Av. Gran Vía del Este, 80, 28031 Madrid, Spain; 23 Hospital Universitario de Jerez, Ctra. Trebujena, s/n, 11407 Jerez de la Frontera, Cádiz Spain; 24 Medical Department Janssen-Cilag, S.A., Madrid, Spain

**Keywords:** Mantle-cell lymphoma, Relapsed/refractory, Ibrutinib, Clinical practice, Real-world evidence

## Abstract

This retrospective study evaluated 66 patients diagnosed with relapsed and/or refractory mantle cell lymphoma (R/R MCL) treated with ibrutinib in Spain in routine clinical practice. At diagnosis, patients had a median age of 64.5 years, 63.6% presented with intermediate/high sMIPI (simplified prognostic index for advanced-stage mantle cell lymphoma), 24.5% had the blastoid variant, and 55.6% had a Ki67 > 30%. Patients had received a median of 2 prior lines of therapy (range 1–2; min–max 1–7). Overall response rate was 63.5%, with 38.1% of patients achieving complete response (CR). With a median duration of ibrutinib exposure of 10.7 months (range 5.2–19.6; min–max 0.3–36), the median progression-free survival (PFS) and overall survival (OS) were 20 months [95% confidence interval (CI) 8.8–31.1] and 32 months (95% CI 22.6–41.3), respectively, and were not reached in patients achieving CR. No grade ≥ 3 cardiovascular toxicity or bleeding was reported. This study supports that treatment with ibrutinib leads to high response rates and favorable survival outcomes in patients with R/R MCL.

**Supplementary Information:**

The online version contains supplementary material available at 10.1007/s12185-022-03367-z.

## Introduction

Mantle-cell lymphoma (MCL) is a rare subtype of B cell non-Hodgkin lymphoma (NHL). In Europe, the incidence rate is approximately 0.5 cases per 100,000 persons-year with most patients aged ≥ 65 years [1]. The prognosis differs greatly between patients with the indolent variant (10% of cases), characterized by splenomegaly, peripheral blood lymphocytosis, and little or no nodal disease, and those with clinical features associated with an aggressive disease course [2]. Despite the improvement of survival over time, the median overall survival (OS) in treatment-naive patients remains limited to 4–5 years [3], and 8–12 in younger and fit patients [4, 5].

No standard therapy for patients with MCL currently exists. Treatment strategies are based on the age of patients and their general condition, but none of the treatment options are curative, excluding allogeneic stem cell transplantation (alloSCT) in some patients. Many patients with MCL are not candidates for intensified regimens typically consolidated with autologous SCT (autoSCT) that significantly improve outcomes in young, fit patients. Furthermore, the benefit of conventional chemoimmunotherapy remains insufficient for many cases, hence, patients require a salvage approach. The possibility of a consolidation with alloSCT is usually reserved for fit patients at high risk of early progression following conventional chemotherapy [6].

The approval of ibrutinib, a first-in-class once-daily Bruton’s tyrosine kinase inhibitor, expanded the current salvage therapy options for relapsed/refractory (R/R) MCL. Ibrutinib provides sustained clinical benefit in patients with R/R MCL, as demonstrated in the extended 3.5 years follow-up from a pooled analysis including three studies (phase II PCYC-1104 [7] and SPARK [8], phase III RAY [9]), leading to median progression-free survival (PFS) and OS of 12.5 and 26.7 months, respectively [10], with better outcomes in patients receiving ibrutinib in the first relapse and those achieving a complete response (CR).

The PFS and OS benefits obtained with ibrutinib in clinical trials have also been observed in the real-world setting [11–16], but the need remains for more real-world studies to reaffirm the use of ibrutinib as standard of care for patients with R/R MCL. In this real-world observational study, we describe the clinical characteristics, management and outcomes of patients with R/R MCL receiving ibrutinib in routine clinical practice in Spain.

## Materials and methods

### Study design and patient population

IBRORS-MCL is a retrospective study conducted at 24 Spanish centers. Patients diagnosed with MCL were included if they had initiated treatment with single-agent ibrutinib for R/R disease between January 2016 (the date of commercialization of ibrutinib in Spain) and up to 6 months before the initiation of the study in September 2018.

The study was approved by the Ethics Committee (EC) of the participating centers and conducted following the Helsinki Declaration and national regulations. Written informed consent was given by all patients, except those who passed away during treatment, for whom a waiver was granted by the EC.

### Data collection and assessments

A retrospective medical record review was performed to collect data on medical history and MCL-related data from diagnosis, including clinical characteristics, comorbidities, concomitant therapies, and therapies used both pre- and post-ibrutinib. The clinical efficacy parameters of ibrutinib included overall response rate (ORR), CR rate, time to response, duration of response (DOR), PFS, OS, and time to subsequent MCL treatment. The safety profile of ibrutinib was also evaluated based on the adverse events (AEs) reported during treatment with ibrutinib, and the dose modifications and treatment discontinuations due to AEs.

### Statistical analysis

Descriptive statistics were used to summarize patient demographics, clinical characteristics, and safety data, including measures of central tendency and dispersion [mean (standard deviation), or median (interquartile range)] for quantitative variables and absolute (*n*) and relative (%) frequencies for qualitative ones.

The ORR was defined as the proportion of patients achieving a CR and partial response (PR) per investigator assessment. Time to initial response was defined from the initiation of ibrutinib to the achievement of CR or PR. DOR was determined from the date on which CR or PR was achieved to the date of disease progression or death, whichever happened first. PFS was measured from the start of ibrutinib to progression or death from any cause and OS was calculated from the start of ibrutinib to death from any cause. Patients were censored at the date of the last available follow-up if still alive or remain without disease progression at the time of the analysis.

Exploratory analyses were performed to assess the outcomes (PFS and OS) according to the achievement of CR or PR. Time-to-event endpoints were estimated using the Kaplan–Meier method, and a log-rank test was used to compare the outcome between subgroups.

Multivariate Cox regression analyses were performed to assess potential prognostic factors for PFS and OS. A multivariate logistic regression analysis was carried out to identify potential predictive response factors. The covariates selected for the analyses included age, ECOG performance status (PS), disease stage, simplified mantle cell lymphoma international prognostic index (sMIPI), histologic variant, Ki67 expression levels, the presence of del(17p)/TP53 mutation at diagnosis, lactate dehydrogenase (LDH) in serum, and POD24, defined as progression of disease within 24 months from the start of the first treatment.

Analyses were performed with the Statistical Package for the Social Sciences version 22.0 (SPSS Inc, Chicago, USA).

## Results

### Patient characteristics

**Fig. 1:**
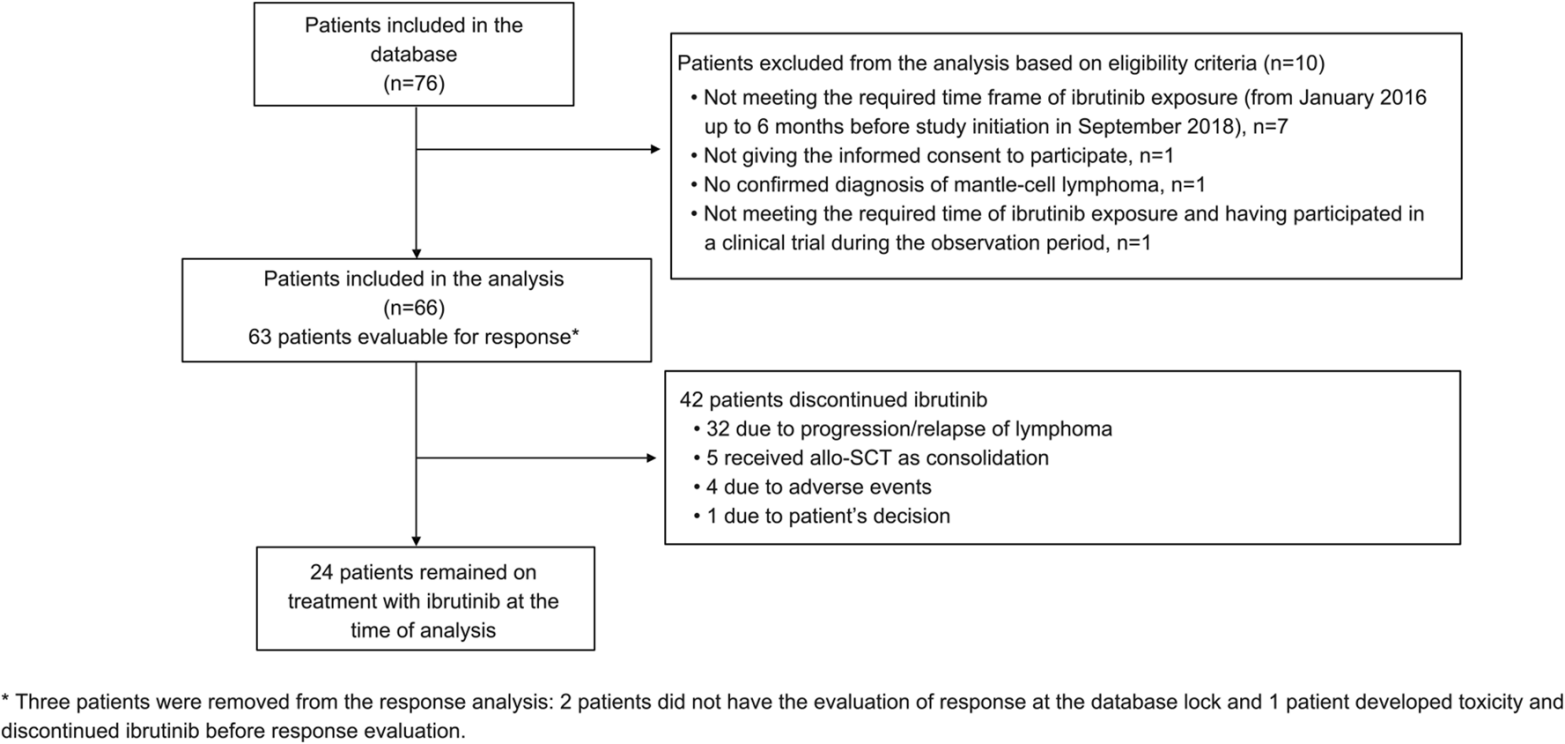
IBRORS-MCL retrospective cohort study flowchart

Between September 2018 and March 2019, 76 patients were included in the study, of whom 66 were evaluable. Ten patients were excluded for not meeting eligibility criteria (Fig. 1). At the time of data collection, 27 patients had died. The median follow-up for survivors was 19.4 months (IQR 13–26.9). The demographic and clinical characteristics are detailed in Table 1. The median age at diagnosis was 64.5 years (range 57–72) and 78.8% of the patients were male. Fifty-nine (93.7%) patients had an ECOG PS of 0–1, 61 (92.4%) patients had stage III–IV disease and 42 (63.6%) had intermediate/high-risk sMIPI (4–10 points). Sixteen patients of 49 (32.6%) presented with an aggressive histology variant (blastoid: *n* = 12, 24.5%, pleomorphic: *n* = 4, 8.2%), and 55.6% of patients (20/36) had a Ki67 level expression > 30%. Two patients of 22 (9.1%) with known del(17p)/TP53 mutation status harbored del(17p)/TP53 mutation.

**Table 1:** Patient characteristics (*n* = 66)

Characteristic	Value
Age, years	64.5 (57–72)
Sex, male	52 (78.8)
ECOG 0–1	59 (93.7)
Stage	
I–II	5 (7.6)
III–IV	61 (92.4)
Histological variant^a^	
Classic and small cell	33 (67.3)
Blastoid	12 (24.5)
Pleomorphic	4 (8.2)
Extranodal involvement	
Bone marrow	47/51 (92.2)
Gastrointestinal	17/44 (38.6)
Central nervous system	4/46 (8.7)
Orbit	3/43 (7.0)
Other sites	20/23 (87.0)
sMIPI	
Low risk (0–3)	24 (36.3)
Intermediate risk (4–5)	26 (39.4)
High risk (6–10)	16 (24.2)
Ki67 > 30%	20/36 (55.6)
Del(17p)/TP53^b^	2/22 (9.1)
Analytical parameters	
Hb, g/dL	12.7 (2.3) (*n* = 61)
Platelets, × 10^3^/µL	186.3 (110.5) (*n* = 61)
Leukocytes, × 10^3^/µL	13.9 (16.5) (*n* = 61)
LDH, U/L	369.3 (255.9) (*n* = 58)
β2-microglobulin, mg/L	3.7 (1.9) (*n* = 42)
Therapy before ibrutinib	
Prior lines of therapy	
Median	2 (range 1–2) (up to 7 lines)
1-2	51 (77.2)
3-4	11 (16.6)
> 4	4 (6.1)
Front-line therapy (*n* = 65)	
R-hyperCVAD	14 (21.5)
R-CHOP/DHAP	9 (13.8)
R-CHOP	12 (18.4)
Autologous SCT consolidation	14 (21.2)
Maintenance rituximab	12 (18.5)
Comorbidities prior to ibrutinib initiation^c^; *n* = 60	
Hypertension	31 (47)
Dyslipidemia	24 (36.4)
Previous neoplasia	18 (27.3)
Cardiovascular disease	14 (21.2)
Diabetes mellitus	14 (21.2)
Obesity	14 (21.2)
Allergy	14 (21.2)

Data are mean (SD), median (range) and n/n assessed (%) unless otherwise specified

Other sites of extranodal involvement included: thyroid (1); testicular (1); spleen (6); amygdala (2); lung (2); liver (4); pleura (2); breast (1); bones (1); cavum (3), lacrimal gland (1)

*ECOG* Easter Cooperative Oncology Group, *LDH* lactate dehydrogenase, *sMIPI* simplified mantle cell lymphoma International Prognostic Index

^a^Available data (*n* = 49)

^b^Percentage calculated on dataset with known del(17p)/TP53 mutation status

^c^Comorbidities presented by < 20% of patients were: gastrointestinal disorders (18.2%), respiratory disorders (12.1%), renal failure (9.1%), liver disorders (9.1%), thrombotic disorders (6.1%), and peripheral (3.3%) and central nervous system disorders (1.7%)

Sixty patients (90.9%) presented comorbidities at the time of ibrutinib initiation, being the most common (affecting > 25%) hypertension (*n* = 31, 47%), dyslipidemia (*n* = 24, 36.4%) and previous neoplasia (*n* = 18, 27.3%) (Table 1). Fourteen patients (21.2%) had cardiovascular disease, including ischemic heart disease (*n* = 6), heart failure (*n* = 4), atrial fibrillation (AF) (*n* = 2) and other arrhythmias (*n* = 3). Forty-six patients (69.7%) received concomitant medication, including antihypertensives (*n* = 30, 45.5%), antiplatelet therapy (*n* = 11, 16.7%) and anticoagulant treatment (*n* = 4, 6.1%).

### Pre-ibrutinib therapy for MCL

The median number of prior lines of therapy for MCL before ibrutinib was 2 (range 1–2) (min–max 1–7), with 51 (77.2%) patients receiving ≤ 2 prior lines. As first line, 65 (98.5%) patients received up-front conventional chemotherapy ± rituximab and one patient received radiotherapy (RT). The most frequent intensive therapies used before ibrutinib were R-hyperCVAD (rituximab and hyperfractionated cyclophosphamide, vincristine, adriamycin and dexamethasone) in 14 (21.5%) patients and R-CHOP/DHAP (alternating courses of cyclophosphamide, doxorubicin, vincristine, prednisone/dexamethasone, cytarabine, cisplatin (CHOP/DHAP) plus rituximab) in 9 (13.8%) patients. The most frequent conventional non-intensive chemotherapy was R-CHOP, administered to 12 (18.4%) patients. Fourteen patients (21.2%) received autoSCT as a first-line consolidation therapy after intensive regimens. Twelve patients received rituximab maintenance (*n* = 4 after intensive and *n* = 8 after conventional non-intensive regimens) (Table 1). After first-line therapy, 12 patients (18.2%) relapsed within the first 2 years (POD24 patients).

Forty-three (65.2%) patients received second-line treatment for MCL other than ibrutinib, mainly with conventional chemoimmunotherapy regimens (*n* = 31), the most frequently used being RB [rituximab plus bendamustine] in 14 patients, R-GemOX [rituximab, gemcitabine and oxaliplatin] in 8 patients and R-BAC [rituximab, bendamustine and cytarabine] in 2 patients). Intensive chemoimmunotherapy with R-hyperCVAD alternating with high-dose methotrexate and cytarabine was given to 4 patients and R-ESHAP (rituximab, etoposide, cisplatin, cytarabine and prednisone) to 5 patients.

Nine patients had a consolidation with SCT (7 autoSCT and 2 alloSCT), and 6 patients received rituximab maintenance after the second-line, none of them after transplantation. Regimens administered in the third line of MCL therapy before ibrutinib to 13 patients were mainly rituximab-containing chemotherapies.

### Ibrutinib therapy

The median age at ibrutinib initiation was 69.3 years (range 60.9–76.2). Ibrutinib was used as a second and third-line therapy in 20 (30.3%) and 31 (47%) patients, respectively, and in later lines in 15 patients (22.7%). The median time from diagnosis of MCL to the start of ibrutinib treatment was 4.2 years (1.2–7.3). At the time of ibrutinib initiation, 12 (18.2%) patients were refractory to the last line of treatment, with half of them being refractory to more than one line of therapy (*n* = 7).

Ibrutinib exposure lasted up to 36 months, with a median duration of 10.7 months (range 5.2–19.6). Twenty-four patients (36.3%) were still in treatment at the time of the analysis. In the response-evaluable population (*n* = 63), ORR was 63.5% (*n* = 40), with 38.1% of patients (*n* = 24) achieving a CR. The median time to initial response was 7.9 months (95% CI 5.9–9.8), and the median DOR was 29.1 months (95% CI 13.1–45.1).

**Fig. 2:**
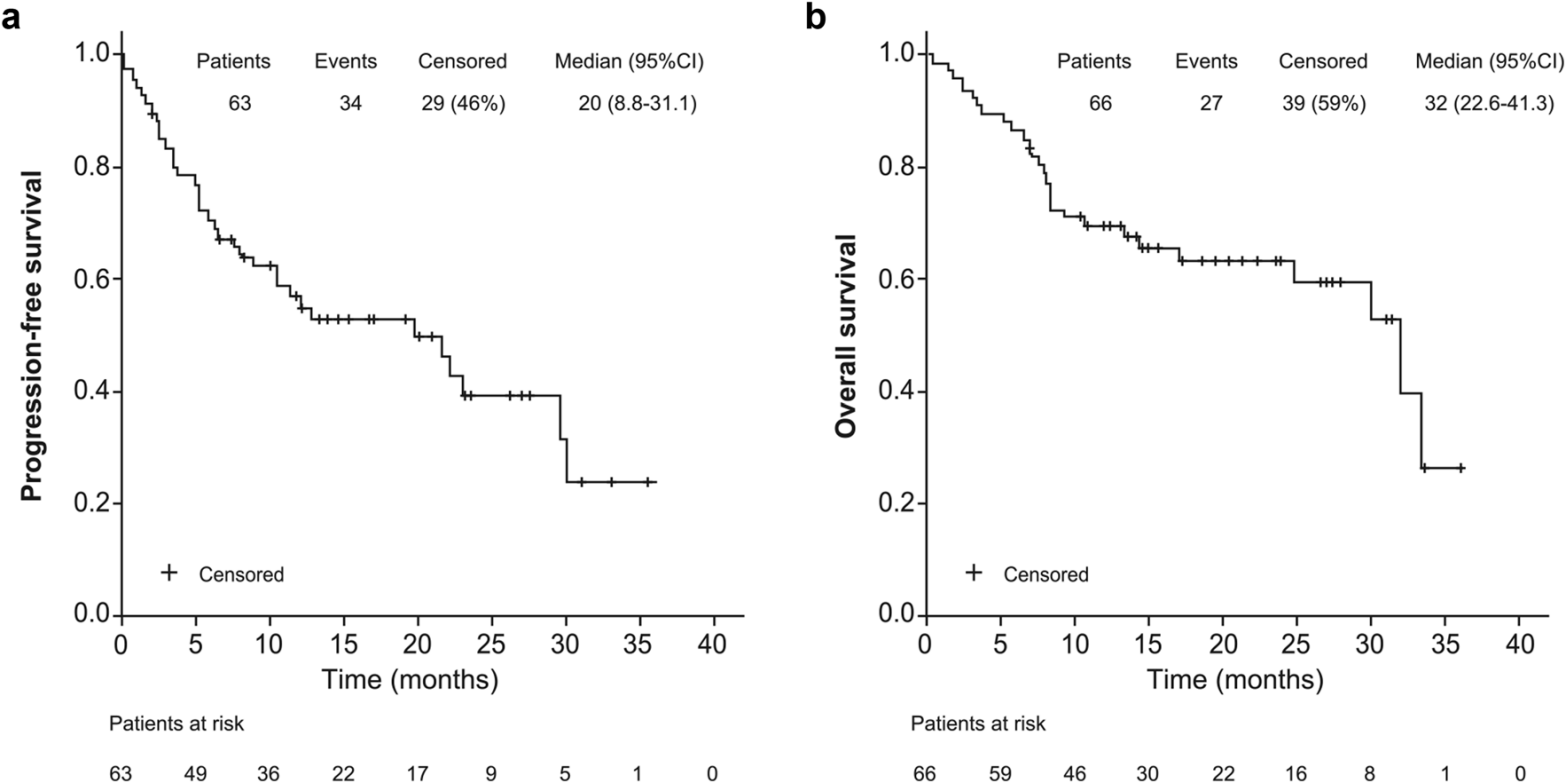
Progression-free survival (**a**) and overall survival (**b**) of R/R MCL patients treated with ibrutinib. *R/R MCL* relapsed and/or refractory mantle cell lymphoma

**Fig. 3:**
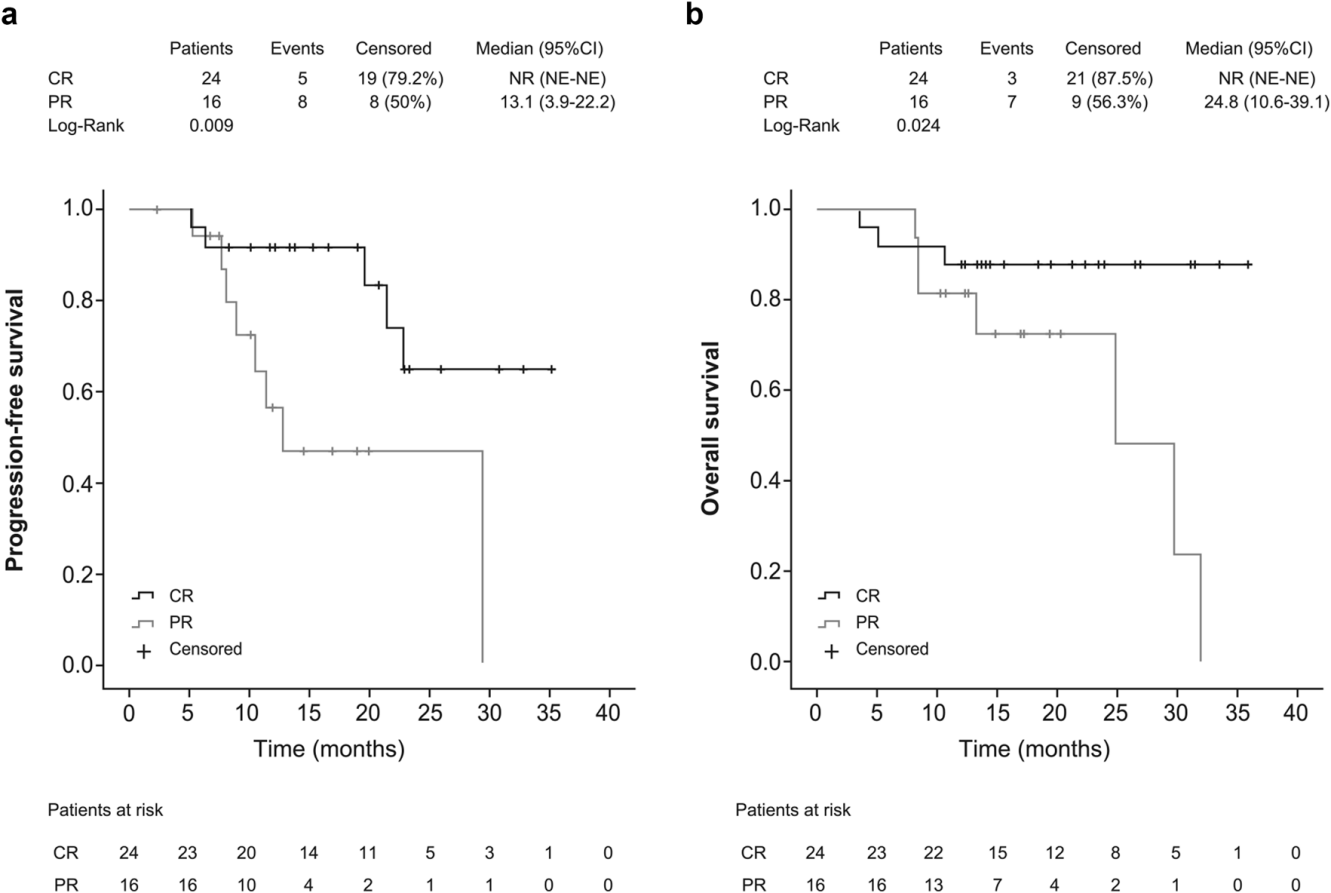
Progression-free survival (**a**) and overall survival (**b**) of R/R MCL patients treated with ibrutinib according to CR vs PR. *R/R MCL* relapsed and/or refractory mantle cell lymphoma, *CR* complete response, *NE* not estimable, *NR* not reached, *PR* partial response

Overall, the median PFS and OS were 20 months (95% CI 8.8–31.1) and 32 months (95% CI 22.6–41.3), respectively (Fig. 2). No significant differences were observed in the median PFS and OS between patients who received ibrutinib in second-line and those treated in later lines (Supplementary Fig. 1). For patients achieving a CR with ibrutinib (38.1%), the median PFS and OS were not reached and were 7.8 months (95% CI 3.6–12.0) and 17 months (95% CI 2.3–31.6), respectively, in patients who failed to achieve a CR. When survival functions were analyzed according to the achievement of a CR or PR, the median PFS and OS were not reached in patients achieving a CR and were 13.1 months (95% CI 3.9–22.2) and 24.8 months (95% CI 10.6–39.1), respectively, in patients achieving a PR (Fig. 3).

### Predictive and prognostic factors for response and clinical outcome

The univariate Cox regression analysis showed that Ki67 level > 30%, the presence at diagnosis of del(17p)/TP53 mutation and high sMIPI were risk factors affecting PFS, although the presence of a high Ki67 level (> 30%) was only retained as an independent prognosis factor for PFS in the multivariate model. A high sMIPI, Ki67 level > 30%, del(17p)/TP53 mutation, and POD24 were identified as risk factors for OS in the univariate COX regression analysis, with the presence of del(17p)/TP53 mutation as the only independent factor associated with a higher risk of decreasing OS in the multivariate model. A high Ki67 level was identified as the only factor independently affecting ORR (Table 2).

**Table 2:** Univariate and multivariate regression models evaluating risk factors for PFS, OS and ORR in R/R MCL treated with ibrutinib

Variable	PFS		OS		ORR	
	Univariate analysis	Multivariate analysis	Univariate analysis	Multivariate analysis	Univariate analysis	Multivariate analysis
Age (≤ 65^a^ vs > 65)	1.31 (0.67–2.58); *p* = 0.42		1.81 (0.82–3.99); *p* = 0.13		0.75 (0.26–2.09); *p* = 0.58	
Stage (IV^a^ vs others)	1.70 (0.59–4.94); *p* = 0.32		2.09 (0.65–6.69); *p* = 0.21		0.18 (0.01–1.84); *p* = 0.14	
ECOG (0–1^a^ vs 2)	1.84 (0.75–4.49); *p* = 0.17		1.63 (0.61–4.38); *p* = 0.32		0.52 (0.11–2.34); *p* = 0.40	
sMIPI (high risk 6–10^a^ vs low–moderate risk: 0–5)	**0.28 (0.13–0.58)**; *p* = **0.001**		**0.36 (0.16–0.82)**; *p* = **0.015**		2.51 (0.77–8.21); *p* = 0.12	
Histology variant (blastoid^a^ vs others)	0.88 (0.36–2.14); *p* = 0.78		0.85 (0.31–2.33); *p* = 0.75		1.20 (0.31–4.60); p = 0.78	
Ki67 (≤ 30^a^ vs > 30%)	**3.09 (1.09–8.78);** *p* =** 0.03**	**3.09 (1.09–8.78)**; *p* = **0.03**	3.84 (0.82–17.87); *p* = 0.08		**0.09 (0.01–0.90); ***p* = **0.04**	**0.09 (0.01–0.90);** *p* = **0.04**
Del (17p)/TP53 (no^a^ vs yes)	**28.53 (2.52–322.34)**; *p* = **0.007**		**10.83 (1.78–65.79);** *p* =** 0.010**	**10.83 (1.78–65.79);** *p* = **0.010**	0.00 (–); *p* = 0.99	
POD24 (no^a^ vs yes)	1.50 (1.00–1.00); *p* = 0.36		**2.57 (1.11–5.96);** *p* = **0.027**		1.00 (0.26–3.89); *p* = 0.99	
LDH	1.00 (1.00–1.00); *p* = 0.11		1.00 (0.99–1.00); *p* = 0.48		1.00 (0.99–1.00); *p* = 0.61	

Data are HR (95%CI) for survival outcomes and OR (95%CI) for ORR

*HR* hazard ratio, *ECOG* Eastern Cooperative Oncology Group, *LDH* lactate dehydrogenase, *PFS* progression-free survival, *ORR* overall response rate, *OR* odds ratio, *OS* overall survival, *POD24* progression of disease within 2 years, *R/R MCL* relapsed and/or refractory mantle cell lymphoma, *sMIPI* simplified prognostic index for advanced-stage mantle cell lymphoma

^a^Chosen as reference category

### Safety

Thirty-six (54.5%) patients reported a total of 63 AEs during treatment with ibrutinib, the majority of which (81%) were grade 1–2 (Table 3). The most frequent grade 1–2 non-hematological AEs were diarrhea in 8 (12.1%) patients, infection in 7 (10.6%) patients and arthromyalgia in 5 (7.6%), and the most frequent grade 1–2 hematological AEs were petechiae/ecchymosis in 8 patients (12.1%) and thrombocytopenia in 5 (7.6%) patients. The most common grade ≥ 3 AEs were infections in 4 (6.1%) patients, neutropenia in 3 (4.5%) patients and ischemic stroke in 2 patients (3%). Fourteen infections occurred in 11 patients during treatment with ibrutinib, mainly bacterial infections (*n* = 4), herpes zoster (*n* = 4) and infections affecting the lower respiratory tract (*n* = 3). One patient experienced a grade 5 infection caused by *Pseudomonas aeruginosa*. No grade 3 atrial fibrillation or bleeding events were reported.

**Table 3:** Adverse events during treatment with ibrutinib in 66 R/R MCL patients

	Grade 1–2	≥ Grade 3	Total
Non-hematological AEs, *n* (%)			
Infection	7 (10.6)	4 (6.1)	11 (16.7)
Diarrhea	8 (12.1)	0	8 (12.1)
Arthromyalgia	5 (7.6)	0	5 (7.6)
Asthenia	3 (4.5)	1 (1.5)	4 (6.1)
Edema	3 (4.5)	0	3 (4.5)
Elevated transaminases	2 (3)	0	2 (3)
Abdominal pain	2 (3)	0	2 (3)
Ischemic ictus	0	2 (3)	2 (3)
Flatulence	1 (1.5)	0	1 (1.5)
Rash	1 (1.5)	0	1 (1.5)
Constipation	1 (1.5)	0	1 (1.5)
Atrial fibrillation	1 (1.5)	0	1 (1.5)
Neoplasia	0	1 (1.5)	1 (1.5)
Other cardiovascular	1 (1.5)	0	1 (1.5)
Weight loss	0	1 (1.5)	1 (1.5)
Hematological AEs, *n* (%)			
Petechiae/ecchymosis	8 (12.1)	0	8 (12.1)
Thrombocytopenia	5 (7.6)	0	5 (7.6)
Neutropenia	2 (3)	3 (4.5)	5 (7.6)
Leucopenia	1 (1.5)	0	1 (1.5)

The data within the table represent the number of patients (%)

*AEs* adverse events, *R/R MCL* relapsed and/or refractory mantle cell lymphoma

Nineteen patients required 22 dose reductions of ibrutinib, mainly due to AEs (9 hematological and 11 non-hematological toxicities), and in half of the cases the dose of ibrutinib was resumed. Forty-two patients discontinued ibrutinib, mainly due to disease progression (*n* = 32, 48.5%), alloSCT (*n* = 5, 7.6%), AEs (*n* = 4, 6%) and one patient’s decision (*n* = 1, 1.5%). The AEs leading to ibrutinib discontinuation were 1 general deterioration, 1 constipation, 1 sepsis caused by *Staphylococcus aureus* and the above-mentioned grade 5 infection by *P. aeruginosa*.

### Post-ibrutinib treatment

Five patients received a consolidation with alloSCT following ibrutinib treatment discontinuation. Of these, 2 patients remained in CR, 1 progressed and 2 patients did not have the response evaluated at the time of analysis. Twenty-five (37.9%) patients received subsequent therapies for MCL after ibrutinib treatment discontinuation [median number of post-ibrutinib therapies received was 1 (range 1–4)].

The first subsequent therapies used after ibrutinib included R-GEMOX-like regimens (*n* = 5; 20%), lenalidomide-based regimens (*n* = 5; 20%), bendamustine-based regimens (*n* = 4; 16%), R-CHOP-like regimens (*n* = 3; 12%), palliative care regimens (*n* = 3; 12%) and others (*n* = 5; 20%).

## Discussion

This retrospective study provides a comprehensive description of the use of ibrutinib in the management of patients with R/R MCL in a real-world setting in Spain. Even though the efficacy and safety of ibrutinib are well documented, the current study supports its effectiveness and good tolerability when used under routine clinical practice conditions, expanding the real-world evidence currently available.

Our series mainly included MCL patients with advanced disease, with more than one extranodal site affected, who had been moderately pre-treated (77% of patients had received 1–2 prior lines of therapy) compared with the patients receiving 2 prior lines of therapy or less in phase II SPARK (52.5%) and phase III RAY (68%) studies [8, 9] and other real-world cohorts including more heavily pre-treated patients (ranging from 55 to 70%) [11, 13, 15].

In this study, the median time from diagnosis to the start of ibrutinib was 4 years and 18% of patients relapsed within 2 years after first-line therapy. This real-world analysis showed that ibrutinib treatment yields a high response rate and favorable outcomes in terms of PFS and OS in patients with R/R MCL. The ORR of 64% is consistent with that obtained in the extended 3.5 years follow-up from the pooled analysis with 370 patients treated in the clinical trial setting (ORR 69.7%) [10] and other real-life retrospective studies by Jeon et al. (64%) [13], Epperla et al. (65%) [12], Yi et al. (64.8%) [16] and McCulloch et al. (69%) [14]. Although not directly comparable to the clinical trial setting, the CR rate observed in the present study (38%) is higher than that observed in the pooled analysis (27%) [10] and similar observational studies (ranging from 15 to 27%) [11, 13, 14]. This is probably due to a larger proportion of patients in our study receiving 1–2 prior lines, which supports previous evidence showing better outcomes when ibrutinib is used in earlier lines of therapy [6, 16] as well as the increased presence of poor prognostic factors with increasing lines of therapy [17]. Lower-risk disease (76% with low-intermediate sMIPI) and better performance in our patients (94% with an ECOG PS 0–1) compared to other real-world cohorts [11, 13, 15] may have also contributed to these observations. Conversely, the proportion of patients with blastoid variant (24%), a well-known adverse prognostic factor, was higher in our cohort than in the clinical trial setting (12%) [10], and real-life Korean (3.4%) [16], Italian (3.9%) [11], and UK cohorts (14–18%) [14, 15].

It is also worth mentioning that the median OS of 32 months is comparable to the one obtained in the clinical trial setting (26.7 months) and that the PFS of 20 months was superior, with similar median treatment exposure [10]. The median PFS and OS we observed are similar to the Korean cohorts published by Jeon et al. (27 and 35 months) [13] and Yi et al. (20.8 and NR) [16], with around 16 months of median treatment exposure, and compare favorably with those from the UK cohorts by Tucker et al. (18.5 and 12 months) and McCulloch et al. (17.8 and 23.9 months) with 10 months of median treatment exposure, and the Italian cohort (12.9 and 16 months) [11], with 6 months of median ibrutinib exposure.

One important finding from this analysis is that the quality of response affected long-term outcomes. In patients achieving a CR, the median PFS and OS were not reached, which is in line with the above-mentioned pooled analysis evaluating ibrutinib in R/R MCL [10], and the new data with up to 7.5 years of extended follow-up [18] where median PFS and OS in the 102 patients (27.6%) with CR were 67.7 months and not reached, respectively, and DOR was 65.6 months, thereby demonstrating highly durable responses in patients achieving a CR with ibrutinib treatment. This would form the basis for the development of effective combination therapies involving ibrutinib [19] with the goal of maximizing CR, as others have previously suggested [17]. In contrast with larger studies, we did not observe significant differences in PFS and OS when analyzed by number of prior lines of therapy. This was probably due to fewer patients receiving ibrutinib in second-line of therapy than in later lines, and to the low number of patients in the present study to perform comparisons per line of treatment compared to the pooled analysis [10].

The main reason for discontinuing ibrutinib was disease progression in nearly 50% of patients, a slightly lower discontinuation rate than that observed in the pooled analysis (59%) [10] and in other real-world analyses (55–69%) [13, 15, 16, 20], which is likely explained by the shorter follow-up in our study. The risk of disease progression with ibrutinib increases with adverse prognostic features such as the presence of del(17p) mutation, high sMIPI score, high Ki67 level, blastoid morphology, and primary refractory disease [10, 13, 21]. In our study, however, we did not find most of these common risk factors significantly impacted clinical outcomes, nor did the early progression of disease in the course of MCL treatment (POD24), as confirmed by others [22], probably due to the number of patients analyzed. Ki67 level was the only independent factor affecting response and PFS, with a high Ki67 level being associated with a higher risk of poor response and disease progression on ibrutinib therapy. The only 2 patients assessed for del(17p)/TP53 mutation in our series precludes any conclusion about the prognostic role of this genetic alteration in OS.

Also in line with previous reports, the tolerability profile of ibrutinib was acceptable. Hematologic and non-hematologic events were mainly grade 1–2 (81%) and grade ≥ 3 events were experienced in less than 20% of patients. Moreover, no grade ≥ 3 cardiovascular toxicity or bleeding events were reported. The low cardiovascular toxicity observed with ibrutinib is remarkable, contrarily to what would be expected in this real-world population with a high percentage of patients with previous cardiovascular diseases. Overall, in this series, with no unexpected safety signals observed, the favorable safety profile of ibrutinib is confirmed.

Patients who progress on ibrutinib seem to have a poor prognosis despite salvage therapy, with median OS ranging from 1.4 to 10 months [20, 21, 23, 24]. The available evidence suggests that the poor post-ibrutinib progression clinical outcome could be attributable to adverse prognostic factors increasing during the evolution of the disease across the different lines of therapy [9]. In clinical practice, there is no established standard of care in the post-ibrutinib setting. The ORR rates achieved after ibrutinib treatment vary substantially depending on whether the regimens used include chemotherapy, PI3K inhibitors, lenalidomide or bortezomib (29–32%) [23–25], venetoclax (53%) [26], or R-BAC (83%) which seems the most effective treatment option to use in the post-ibrutinib setting to date [20]. In this study, the five patients who were consolidated with alloSCT add to the evidence suggesting the effectiveness of using ibrutinib as a bridge to alloSCT in R/R MCL [27]. Recently, CAR-T cell therapy has proven to be a promising approach, inducing durable responses in patients with R/R MCL after failure to respond to BTK inhibitor therapy [28, 29].

This study has several limitations, including the retrospective nature of the study, the lack of central response evaluation and the fact that the study was not powered for the exploratory uni- and multi-variable analysis performed. Despite these limitations, the results from this study are in line with previously published clinical trials and real-world studies, and show the benefits of ibrutinib in R/R MCL in clinical practice.

In summary, the IBRORS-LCM study supports the use of ibrutinib for the treatment of patients with R/R MCL, as it shows good response rates and survival outcomes in these patients, with improved outcomes among those achieving a CR. Notably, treatment with ibrutinib in our patients did not result in additional and unexpected toxicities when compared to those found in previous studies.

## Supplementary Information

Below is the link to the electronic supplementary material.

Supplementary file1 (EPS 2260 KB) Progression-free survival a and overall survival b according to prior lines of therapy
